# Vascular dysfunction in sporadic bvFTD: white matter hyperintensity and peripheral vascular biomarkers

**DOI:** 10.1186/s13195-024-01422-x

**Published:** 2024-04-05

**Authors:** Min Chu, Deming Jiang, Haitian Nan, Lulu Wen, Li Liu, Miao Qu, Liyong Wu

**Affiliations:** Department of Neurology, Xuanwu Hospital, Capital Medical University, Beijing, 100053 China

**Keywords:** Vascular factor, Frontotemporal dementia, Neurodegeneration, Clinical measures

## Abstract

**Background:**

Vascular dysfunction was recently reported to be involved in the pathophysiological process of neurodegenerative diseases, but its role in sporadic behavioral variant frontotemporal dementia (bvFTD) remains unclear. The aim of this study was to systematically explore vascular dysfunction, including changes in white matter hyperintensities (WMHs) and peripheral vascular markers in bvFTD.

**Methods:**

Thirty-two patients with bvFTD who with no vascular risk factors were enrolled in this cross-sectional study and assessed using positron emission tomography/magnetic resonance (PET/MRI) imaging, peripheral plasma vascular/inflammation markers, and neuropsychological examinations. Group differences were tested using Student’s *t*-tests and Mann–Whitney U tests. A partial correlation analysis was implemented to explore the association between peripheral vascular markers, neuroimaging, and clinical measures.

**Results:**

WMH was mainly distributed in anterior brain regions. All peripheral vascular factors including matrix metalloproteinases-1 (MMP-1), MMP-3, osteopontin, and pentraxin-3 were increased in the bvFTD group. WMH was associated with the peripheral vascular factor pentraxin-3. The plasma level of MMP-1 was negatively correlated with the gray matter metabolism of the frontal, temporal, insula, and basal ganglia brain regions. The WMHs in the frontal and limbic lobes were associated with plasma inflammation markers, disease severity, executive function, and behavior abnormality. Peripheral vascular markers were associated with the plasma inflammation markers.

**Conclusions:**

WMHs and abnormalities in peripheral vascular markers were found in patients with bvFTD. These were found to be associated with the disease-specific pattern of neurodegeneration, indicating that vascular dysfunction may be involved in the pathogenesis of bvFTD. This warrants further confirmation by postmortem autopsy. Targeting the vascular pathway might be a promising approach for potential therapy.

## Background

Recently, researchers discovered that vascular dysfunction was involved in the frontotemporal dementia (FTD) neurodegeneration process. One single-nucleus RNA sequence study targeting the brain cortex of 13 FTD-*GRN* patients showed severe neurovascular dysfunction and perturbed blood–brain barrier (BBB), posing interest in the vascular function of FTD [1]. White matter hyperintensity (WMH) can reflect neurovascular damage in vivo and typically appears in cerebral small vessel disease or hypertension [2]. WMH has primarily been reported in FTD patients who carried *GRN* mutations without vascular factors [3]. However, the WMH in sporadic FTD remains unknown. One study demonstrated WMH in a mixed genetic and sporadic FTD group and showed that it was associated with disease severity, independent of vascular risk factors, suggesting that vascular factors might play important roles in sporadic FTD [4].

Some peripheral cytokines frequently studied in cardiovascular disease such as matrix metalloproteinase-1 (MMP-1), matrix metalloproteinase-3 (MMP-3), osteopontin, and pentraxin-3 can be used as markers to reflect vascular inflammation, endothelial function, or permeability of the BBB [5–9]. The abnormalities of these markers can provide in vivo evidence for vascular dysfunction of FTD. However, the correlation between vascular dysfunction and brain changes, inflammation, and clinical measures and whether vascular dysfunction exists in the early stages of disease in sporadic bvFTD are still unclear.

In this study, patients with sporadic bvFTD and healthy controls with no vascular risk factors were enrolled. Plasma samples, hybrid positron emission tomography/magnetic resonance imaging (PET/MRI) scans, and neuropsychological scales were collected. We aimed to systematically explore the vascular dysfunction process in FTD. We hypothesized that (1) vascular dysfunction exists in sporadic bvFTD with central and peripheral vascular marker abnormalities; and (2) vascular dysfunction of sporadic bvFTD is associated with gray matter (GM) changes, inflammation, and clinical outcomes.

## Methods

### Ethics

This cross-sectional study was conducted in line with the Declaration of Helsinki. Our research protocols were approved by the institutional review board and the ethics committee of Xuanwu Hospital. Related guidelines and regulations for the use of human subjects in research were followed in this study. We obtained written informed consent from the subjects or their guardians before the initiation of the research.

### Participants

Sixty-four right-handed subjects, including 32 bvFTD patients and 32 normal controls (NCs), were enrolled in our study between July 1, 2014, and October 31, 2021, at Xuanwu Hospital. All patients in the bvFTD group met the diagnosis of probable bvFTD according to the 2011 bvFTD consensus criteria [10]. Age- and sex-matched healthy controls were enrolled, with no complaints of cognitive dysfunction or behavior deficits, as well as a normal score range in neuropsychological tests (Mini-Mental State Examination [MMSE] score greater than 28 and Frontotemporal Lobar Degeneration Clinical Dementia Rating Scale [FTLD-CDR] score of 0). Participants who had neuropsychiatric diseases such as cerebrovascular disorder, schizophrenia, substance abuse, alcoholism, or tumors were excluded. There was no hypertension, hypercholesterolemia, diabetes, smoking habit, or previous vascular disease found in patients or controls. Body mass index (BMI) was in the normal range (18.5–23.9 kg/m^2^).

### Neuropsychological tests

A standardized neuropsychological assessment was conducted for all participants. The MMSE scale was used to test global cognitive status. The CDR® plus NACC FTLD-SB, scored using the sum of the six domains of the CDR plus the behavior/comportment/personality and language domains, was used to assess FTD disease severity. The Frontal Behavior Inventory (FBI) was used to test the severity of behavioral abnormality. The first 12 items measured negative apathy symptoms (FBI apathy subscale), and the last 12 items measured positive disinhibition symptoms (FBI disinhibition subscale). The Trail Making Test (TMT) was used to evaluate executive function. The Boston Naming Test (BNT) was used to test for language deficits. The Activities of Daily Living (ADL) scale was used to test activities of living.

### Vascular and inflammatory factor analysis

We collected peripheral blood from all subjects in acid-citrate dextrose Vacutainers (BD Biosciences, San Jose, CA, USA). Plasma was obtained from blood by centrifugation at 2100 rpm for 10 min. Then, the plasma was separated, aliquoted, and stored at − 80 °C for further cytokine analysis. A panel of four cytokines (MMP-1, MMP-3, osteopontin, and pentraxin-3) was measured in the plasma for Bio-plex Pro human inflammation panel (Bio-Rad, 17008653, Hercules, CA, USA) detection. We ran one sample per subject at one time, in line with the operating protocol. First, we incubated a 25-μl plasma sample with antibody-coupled fluorescent beads. Second, we washed and incubated the sample with biotinylated detection antibodies detected by streptavidin–phycoerythrin. Third, a flow-based Luminex 100 suspension array system (Bio-plex 200; Bio-Rad Laboratories, Inc.) was used to analyze the beads. To determine the sample concentration, standard curves were generated by Bio-plex Manager software, and in the kit, the reference cytokines were provided by the manufacturer. Other inflammation cytokines (tumour necrosis superfamily member 13B [TNFSF/BAFF], IL-29/interferon [IFN]-λ2, IL-32, TWEAK/TNFSF12, and sCD30/TNFRSF8, IFN-γ, IL-10, IL-12p70, IL-17A, IL-1β, IL-2, IL-4, IL-6, and TNF-α) were assessed with the same method in our previous research [11].

### Neuroimaging analysis

#### PET/MRI acquisition parameters

A hybrid 3.0-T PET/MRI scanner (SIGNA PET/MR, GE Healthcare, WI, USA) was used to acquire the images. A vendor-supplied 19-channel union coil of head and neck was used to acquire the PET and MRI data simultaneously. After applying 3.7 MBq/kg ^18^F-FDG for each subject, three-dimensional T1-weighted (3D-T1) sagittal images and ^18^F-fluorodeoxyglucose PET (^18^F-FDG PET) images were acquired in the same session.

The parameters of the multimodal image data were as follows:

1. *3D-T1:* repetition time (TR) = 6.9 ms, echo time (TE) = 2.98 ms, flip angle = 12°, inversion time = 450 ms, matrix size = 256 × 256, field of view (FOV) = 256 × 256 mm^2^, slice thickness = 1 mm, 192 sagittal slices with no gap, voxel size = 1 × 1 × 1 mm^3^, and acquisition time = 4 min 48 s.
2. *T2-weighted fluid-attenuated inversion recovery (FLAIR):* matrix size = 512 × 512, voxel resolution of 0.5 × 0.5 × 6 mm with 6 mm gap, 24 slices, TR = 11 ms, TE = 118 ms.
3. *Static *^*18*^*F-FDG-PET:* matrix size = 192 × 192, FOV = 350 × 350 mm^2^, and pixel size = 1.82 × 1.82 × 2.78 mm^3^, including corrections for random coincidences, dead time, scatter, and photon attenuation. Attenuation correction was performed based on MR imaging of the brain (Atlas-based co-registration of 2-point Dixon).

#### Structural image preprocessing

Computational anatomy toolbox 12 (CAT12), based on statistical parametric mapping 12 (SPM12), was used to preprocess the data. First, MRICRON software was used to convert the DICOM to nifti files. Default settings in the CAT12 toolbox and the “East Asian Brains” ICBM template were used to perform voxel-based morphometry (VBM) preprocessing. 3D T1-weighted images were divided into GM, white matter (WM), and cerebrospinal fluid (CSF) parts. Second, diffeomorphic anatomical registration was used through exponentiated lie algebra normalization to high-dimensionally registered and normalized GM and WM parts of each participant from native space to standard Montreal Neurological Institute (MNI) space. An 8-mm full-width half-maximum Gaussian kernel was used to smooth the images.

#### White matter hyperintensity (WMH) segmentation

Segmentation of WMH was performed from the T2 FLAIR images using the Lesion Segmentation Toolbox (version 3.0.0) in SPM12 [12]. WMH lesion maps was binarized at a probability of 0 and then registered to the MNI space. To obtain the registration transformation matrix, T2 FLAIR images were automatically registered to the native T1-weighted MRI, and then, MRIs were registered to the MNI space. The accuracy of co-registration was confirmed visually, and no subject was excluded due to poor scan quality or inadequate segmentation.

#### PET image preprocessing

SPM12, which was implemented in MATLAB (MathWorks, Natick, MA, USA), was used to preprocess the PET images. For PET spatial normalization, T1-weighted image spatial normalization was applied to the co-registered PET images after the normalization of the structural MRI images. An 8-mm full-width half-maximum isotropic Gaussian kernel was then used for image smoothing. Finally, to create standardized uptake value ratio (SUVR) images, a cerebellum reference region was used to normalize PET images.

#### Regions of interest analysis

For further partial correlation analysis, an atlas-based ROI analysis was conducted to extract the GM volumes and glucose SUVR of 90 brain regions in structural and PET images using the Automated Anatomical Labeling (AAL) atlas. Total WMH volume was extracted from WM lesion maps in native space. The volume of WMH in the frontal, temporal, parietal, occipital, and limbic lobes and subcortical regions were extracted using the standard atlas from the WFU PickAtlas toolbox in the MNI space [13]. FTD-specific GM volume was computed from AAL-maps, including the frontal, temporal, limbic, and basal ganglia regions, and added as covariates in further WMH analysis.

### Statistical analysis

All statistical analyses were performed in SPSS Statistics Version 22 (IBM Corporation, Armonk, NY, USA). All tests were two-tailed with the alpha value set to *p* < 0.05. To compare the group differences, normally distributed data were analyzed using a student’s *t*-test, and skewed data were analyzed using a Mann–Whitney U test. Partial correlation analysis was implemented with age, sex, and education as covariates to explore the association between peripheral vascular markers, inflammatory markers, neuroimaging, and clinical measures. Considering the influence of brain atrophy and clinical severity, when we conducted a partial correlation analysis of WMH and four blood vascular markers, correcting for age, sex, education, frontal, and temporal brain volume and CDR® plus NACC FTLD-SB. The family-wise error (FWE) and false discovery rate (FDR) correction were used in neuroimaging analysis**.**

## Results

### Demographic and clinical features

**Fig. 1:**
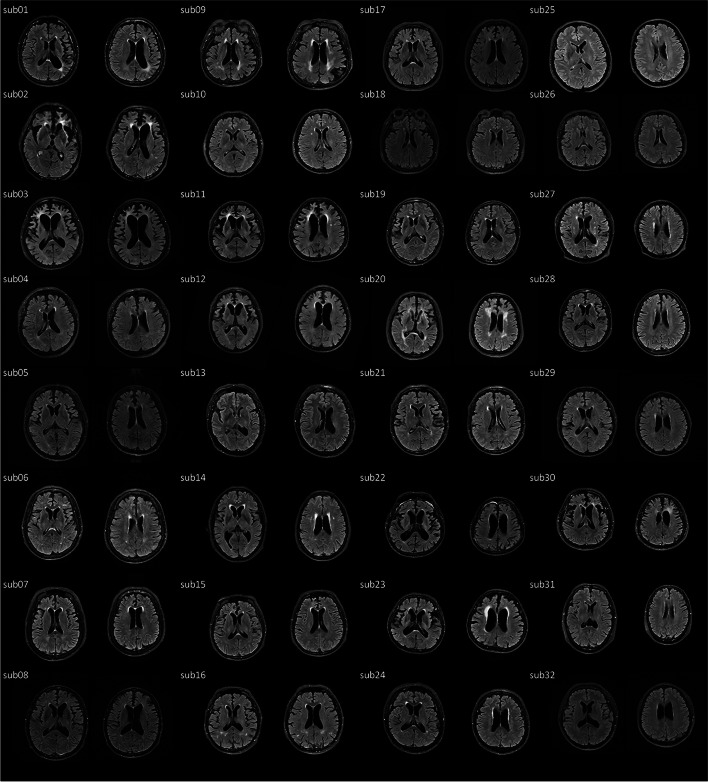
Raw images of white matter hyperintensity (WMH) in patients with FTD. In the raw images of 32 patients with FTD, the WMHs was observed in periventricular in T2 Fluid-Attenuated Inversion Recovery (FLAIR) images. It is more prominent in the anterior brain area, and some affect the posterior brain area

Demographic and clinical features were shown in Table 1. No significant difference was found in age or gender between the bvFTD and NC groups. Patients with bvFTD showed significant impairment in executive function compared with the NC group (TMT-A, 114.72 ± 36.12 vs. 45.63 ± 11.30, *p* < 0.001; TMT-B, 232.19 ± 87.22 vs. 65.71 ± 21.88, *p* < 0.001). Patients with bvFTD also showed symptoms of apathy (FBI apathy, 15.09 ± 7.88) and disinhibition (FBI disinhibition, 6.00 ± 5.07). Raw data of WMH was shown in Fig. 1. The WMHs was observed in periventricular on T2 FLAIR images.

**Table 1:** Demographics and neuropsychological scales in patients with behavioral variant frontotemporal dementia (bvFTD) and normal controls (NCs)

	bvFTD (*n* = 32)	NC (*n* = 32)	***p***
Age (years)	62.81 ± 7.16	60.00 ± 8.52	0.1579
Gender (F/M)	20/12	20/12	1
Years of education	10.78 ± 3.99	11.78 ± 2.90	0.2561
Age at onset (years)	61.09 ± 7.41		
Disease duration (m)	23.81 ± 15.19		
Neuropsychological scale			
MMSE	17.69 ± 5.64	28.72 ± 0.92	< 0.001
TMT-A	114.72 ± 36.12	45.63 ± 11.30	< 0.001
TMT-B	232.19 ± 87.22	65.71 ± 21.88	< 0.001
BNT	18.47 ± 7.06	25.78 ± 2.20	< 0.001
FBI total	21.09 ± 10.55	0	< 0.001
FBI apathy	15.09 ± 7.88	0	< 0.001
FBI disinhibition	6.00 ± 5.07	0	< 0.001
ADL	32.91 ± 11.46	0	< 0.001
CDR® plus NACC FTLD-SB	9.91 ± 5.18	0	< 0.001
BEHAV	1.63 ± 0.71	0	< 0.001
LANG	0.58 ± 0.53	0	< 0.001

*Abbreviations*: *bvFTD* behavioral variant frontotemporal dementia, *MMSE* Mini-Mental State Examination, *CDR® plus NACC FTLD-SB* the sum of the six domains of the CDR plus the behavior/comportment/personality and language domains, *BEHAV* behavior/comportment/personality, *LANG* language, *TMT* Trail Making Test, *FBI* Frontal Behavior Inventory, *BNT* Boston Naming Test, *ADL* activities of daily living scale

### Central and peripheral vascular dysfunction

#### Brain distribution of white matter hyperintensity in bvFTD

**Fig. 2:**
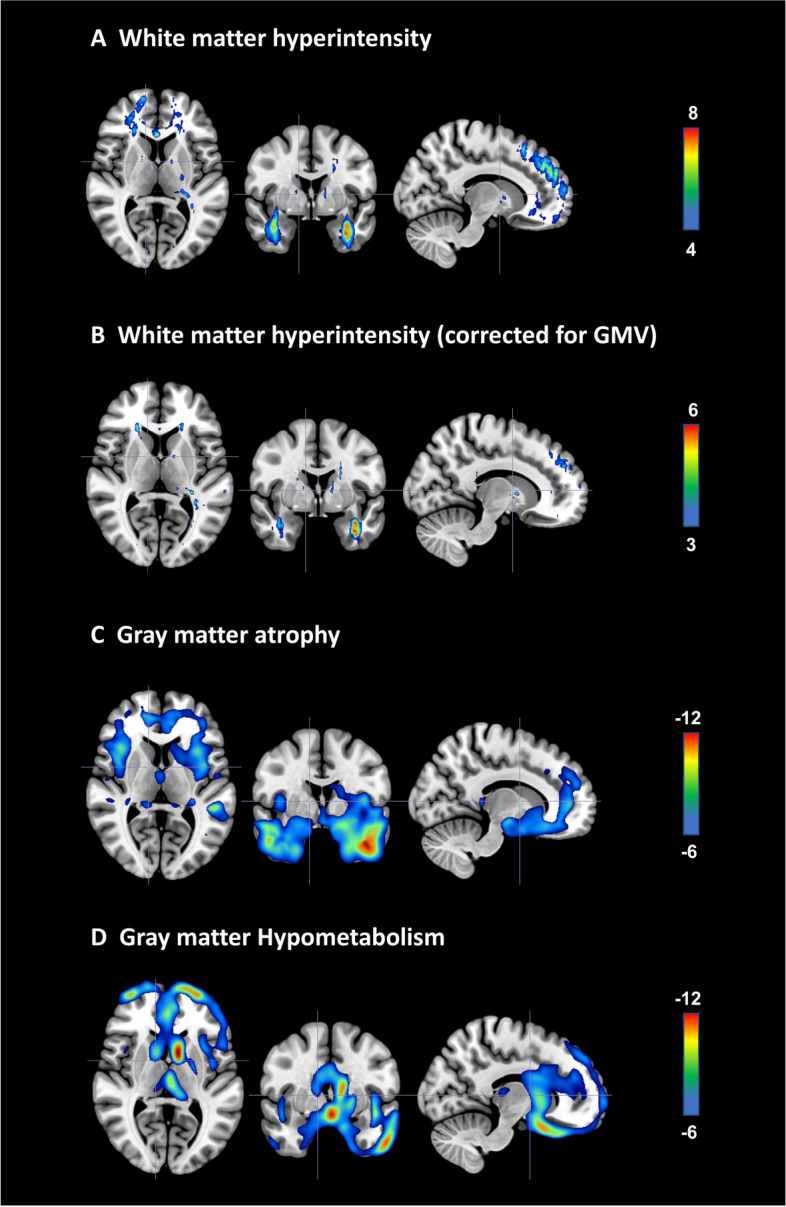
Group comparison of white matter hyperintensity. **A** Increased white matter hyperintensity in behavioral variant frontotemporal dementia (bvFTD), adjusted for age, gender, and years of education, and total intracranial volume (TIV), FDR corrected. **B** Increased white matter hyperintensity in bvFTD remains in anterior brain regions after adjusting for age, gender, years of education, TIV, and FTD-specific gray matter volume, FDR corrected. **C** Fronto-temporal lobe atrophy in bvFTD, adjusted for age, gender, and years of education and TIV, FWE corrected. **D** Fronto-temporal lobe hypometabolism in bvFTD, adjusted for age, gender, and years of education and TIV, FWE corrected

White matter hyperintensity (WMH) was mainly distributed in the anterior brain regions including superior corona radiata and superior longitudinal fasciculus (Fig. 2A). The WMH still remained after correcting the FTD-specific gray matter volume (Fig. 2B).

The distribution of WMH was similar to GM atrophy (Fig. 2C) and hypometabolism (Fig. 2D), which were also mainly distributed in frontotemporal regions.

#### Increased peripheral vascular factors in bvFTD

**Fig. 3:**
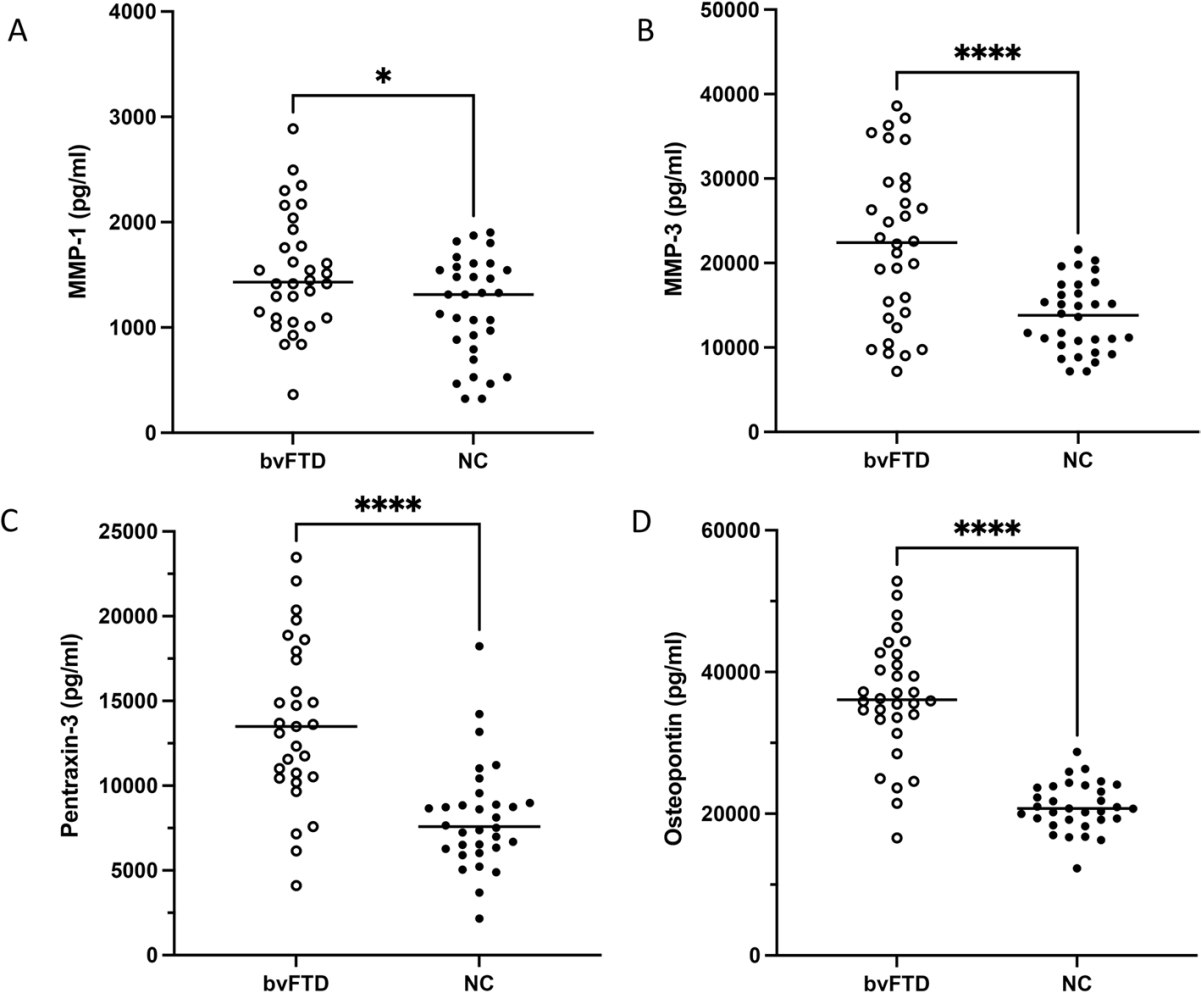
Group comparison of peripheral vascular factors between bvFTD and NC. Levels of MMP-1 (**A**), MMP-3 (**B**), pentraxin-3 (**C**), and osteopontin (**D**) were increased in the bvFTD group compared with controls. Abbreviation: MMP-1, matrix metalloproteinase-1; MMP-3, matrix metalloproteinase-3

All peripheral vascular factors increased in the bvFTD group (Fig. 3), including MMP-1 (1521.69 ± 555.50 vs. 1184.98 ± 479.07 pg/ml, *p* = 0.0117), MMP-3 (22197.39 ± 9471.49 vs. 13645.05 ± 4158.20 pg/ml, *p* < 0.001), osteopontin (36360.30 ± 8301.06 vs. 20977.50 ± 3402.69 pg/ml, *p* < 0.001), and pentraxin-3 (16836.01 ± 11272.09 vs. 8110.22 ± 3127.23 pg/ml, *p* < 0.001).

#### Association between white matter hyperintensity and peripheral vascular marker

The WMH in sub-lobar brain regions was positively associated with the levels of pentraxin (Table 2; R = 0.4138, *p* = 0.0162, FDR adjusted *p* = 0.0521).

**Table 2:** Correlation analysis between white matter hyperintensity (WMH) and peripheral vascular/inflammation markers and clinical parameters in bvFTD

WMH	Others	R	*p*	FDR adjusted *p*
**Frontal**	IL-6	0.4472	0.0103	0.0948
	TNFa	0.5156	0.0025	0.0381^*^
	MMSE	− 0.3598	0.0431	0.2378
	TMT-B	0.3867	0.0288	0.1925
	IADL	0.4938	0.0041	0.0509
	ADL	0.4740	0.0061	0.0450^*^
	CDR® plus NACC FTLD-SB	0.3975	0.0243	0.1698
	BEHAV	0.4999	0.0036	0.0484^*^
**Sub-lobar**	Pentraxim	0.4138	0.0162	0.0521
	TMTB	0.4848	0.0049	0.0556
**Limbic**	IL-6	0.3719	0.0361	0.2144
	TNFa	0.5520	0.0011	0.0190^*^
	TMTB	0.4914	0.0043	0.0520
	IADL	0.3935	0.0259	0.1842
	ADL	0.3758	0.0341	0.1328
	CDR® plus NACC FTLD-SB	0.3781	0.0328	0.2081
	BEHAV	0.3828	0.0306	0.2022

Adjusted for age, sex, and years of education

*Abbreviations*: *bvFTD* behavioral variant frontotemporal dementia, *MMSE* Mini-Mental State Examination, *CDR® plus NACC FTLD-SB* the sum of the six domain scores of the CDR plus the behavior/comportment/personality and language domains, *BEHAV* behavior/comportment/personality, *LANG* language, *TMT* Trail Making Test, *ADL* activities of daily living scale

* of significance

#### Associations of white matter hyperintensity and peripheral inflammation markers/clinical measures

As shown in Table 2, the WMH in the frontal lobe was positively associated with peripheral inflammation (IL-6 and TNFa). It was also negatively associated with MMSE, and positively associated with TMT-B, IADL, ADL, the behavior module of FTLD-CDR, and FTLD-CDR sum. The WMH in sub-lobar brain regions was positively associated with executive function (TMT-B). The WMH in limbic regions was positively associated with peripheral inflammation (IL-6 and TNF a), disease severity including TMTB, IADL, ADL, the behavior module of FTLD-CDR, and FTLD-CDR sum.

After FDR correction, the correlation between WMH in the frontal lobe and TNFa, ADL, and FTLD-CDR behavior modules still existed. The positive correlation between WMH in limbic regions and TNFa also remained.

#### Correlation analysis between peripheral vascular factors and gray matter changes

The plasma level of MMP-1 was negatively correlated with the GM metabolism of the frontal, temporal, insula, and basal ganglia brain regions. The detailed information is presented in Table 3. No other significant associations were found in other cytokines and GM volume/metabolism.

**Table 3:** Partial correlation analysis between MMP-1 and brain metabolism in bvFTD

Vascular factor	Brain regions	R	*p*	FDR-adjust *p*
**MMP-1**	Right inferior frontal	-0.4452	0.0107	0.0168^*^
	Right inferior frontal orbital	-0.3864	0.0289	0.0421^*^
	Right insula	-0.4332	0.0133	0.0205^*^
	Left middle cingulate	-0.4402	0.0117	0.0182^*^
	Right middle cingulate	-0.4538	0.0091	0.0144^*^
	Left cingulate	-0.4879	0.0046	0.0078^*^
	Right cingulate	-0.5160	0.0025	0.0044^*^
	Left hippocampus	-0.3842	0.0299	0.0433^*^
	Right hippocampus	-0.4561	0.0087	0.0139^*^
	Left para-hippocampus	-0.4060	0.0211	0.0315^*^
	Right para-hippocampus	-0.4484	0.0101	0.0159^*^
	Left amygdala	-0.4396	0.0118	0.0184^*^
	Right amygdala	-0.5036	0.0033	0.0057^*^
	Right caudate	-0.4003	0.0232	0.0343^*^
	Right thalamus	-0.4802	0.0054	0.0090^*^
	Right superior temporal	-0.4644	0.0074	0.0120^*^
	Right middle temporal	-0.4574	0.0085	0.0136^*^
	Right superior frontal	-0.3636	0.0408	0.0576
	Right inferior frontal opercular part	-0.3705	0.0369	0.0525

Adjusted for age, sex, and years of education

*Abbreviation*: *MMP-1* matrix metalloproteinase-1

* of significance

#### Associations of peripheral vascular factors and peripheral inflammation markers/clinical measures

Peripheral vascular markers were associated with several peripheral inflammation markers (Table 4; MMP-3 and sCD30/TNFRSF8, osteopontin and BAFF, osteopontin and sCD30/TNFRSF8). After FDR correction, only a correlation between osteopontin and sCD30/TNFRSF8 remained. No other significant correlations were found in clinical measures.

**Table 4:** Positive correlation between peripheral vascular and inflammation markers in bvFTD

Peripheral vascular marker	Inflammation marker	R	*p*	FDR adjusted *p*
**MMP-3**	sCD30/TNFRSF8	0.3673	0.0386	0.2216
**Osteopontin**	BAFF	0.5144	0.0031	0.3959
	sCD30/TNFRSF8	0.6276	0.0001	0.0031^*^

Adjusted for age, sex, and years of education

*Abbreviation*: *MMP-3* matrix metalloproteinase-3

* of significance

## Discussion

This study selected a group of early-onset sporadic bvFTD patients with no vascular risk factors and demonstrated vascular impairment in FTD. This impairment can be manifested in WMH and peripheral vascular marker abnormalities, which are associated with disease-specific GM changes, inflammation, and clinical measures. These results provide novel in vivo evidence that vascular dysfunction and disturbed BBB exist in sporadic bvFTD and are associated with the disease’s pathophysiological process.

WMH has been reported in neurodegenerative disease in recent research. In FTD, WM damage was primarily observed in patients with GRN and accumulated over time [14] because progranulin plays a key role in regulating wound repair and inflammation [15]. Our study extends the evidence for WMH not only in GRN but also in sporadic FTD. In a mixed group of genetic and sporadic FTD patients, WMH and cortical damage were correlated and existed independently [16]. In this study, consistent with previous findings in FTD, we also observed this phenomenon in a group of sporadic bvFTD patients, such that WMH showed a spatial distribution similar to GM atrophy and hypometabolism in the frontotemporal regions. After correction for cortical atrophy, the difference in WMHs remained.

Chronic ischemic conditions in white matter can lead to demyelinating changes that are visible as high signal areas adjacent to the ventricles or subcortically on T2 FLAIR imaging, related to vascular injury, and are effective indicators of vascular dysfunction [2]. The causes of WMH in neurodegenerative disease remains unclear. Recent research has suggested that WMH in AD is associated with neurodegenerative changes as well as parenchymal and vascular amyloid deposition, but not with systemic vascular risk factors [17]. A review also mentioned the relationship between WMH and Aβ pathology in AD was mixed, with posterior brain regions associated with AD pathology, while the anterior is linked to both small vessel disease and AD pathology [18, 19]. Therefore, we speculate that WMHs in FTD might not only be due to the damage of vascular structures by pathological protein deposits but could also result from Wallerian degeneration [20], inflammation, or even methodological issues (image postprocessing). The anterior and posterior high signal white matter may have different underlying mechanisms. A previous study investigating cortical single nucleus RNA sequencing in 13 FTD-*GRN* patients revealed severe neurovascular dysfunction and blood–brain barrier (BBB) disruption [1], which indicates that autopsy studies on sporadic FTD should also be conducted in the future to clarify the vascular issues.

This study was the first to identify a correlation between central WMH and peripheral vascular factors in sporadic bvFTD patients, suggesting the reliability of vascular damage and the potential of peripheral vascular factors as a more convenient and measurable biomarker for predicting central vascular damage. Additionally, inflammation was confirmed as an important pathway in FTD [11, 21]. This study further identified a positive correlation between WMH/peripheral vascular markers and inflammatory markers; we speculate that a bidirectional relationship may exist between inflammation and vascular damage. Further investigation and validation through inflammatory factor testing in CSF are warranted.

A more targeted assessment of functional and behavioral symptoms was conducted in our group of bvFTD patients, and the results showed that WMH in regions such as the frontal and parietal lobes was associated with the severity of executive dysfunction, impairment of daily living abilities, and behavioral abnormalities. The cortical-basal-cortical neural circuits composed of the frontal, temporal, and limbic lobes are known to be impaired in FTD and closely related to the severity of executive dysfunction and behavioral abnormalities [22], which can significantly impact daily living abilities. The correlations between WMH and clinical measures provide more evidence for the role of WMH in the pathological process of FTD.

The four markers we selected regulate abnormal angiogenesis and vascular remodeling, which are well-known in tumors, arthritis, atherosclerosis, restenosis, or hypertension [5, 8, 9]. Matrix metalloproteinases (MMPs) are inflammatory mediators regulating vascular dysfunction, and their changes in FTD are variable. MMP-10 levels were reported to increase in patients with FTD [23]. MMP-2 and MMP-9 were higher in induced pluripotent stem cell (iPSC) lines from patients with *MAPT* mutation [24]. However, another study found that MMP-1, MMP-2, and MMP-9 levels were normal in FTD [25]. MMP-3 was first tested in FTD in this study, and it is known to regulate the BBB permeability via the extracellular regulated protein kinases (ERK) signaling pathway. The MMP-3 abnormalities in bvFTD indicate a perturbance in the BBB [6]. Osteopontin and pentraxin-3 have a two-faced phenotype and dual character that have frequently been reported in cardiovascular disease [9, 26]. The increased marker levels provide evidence for vascular dysfunction and damage to the BBB in FTD.

Vascular dysfunction was associated with prefrontal-anterior cingulate-temporal cortex hypometabolism, which is the typical FTD disease-vulnerable degeneration pattern, indicating a reliable effect of the neurovascular pathway in FTD. The prefrontal, anterior cingulate, and temporal pole are key nodes of the salience network that are frequently damaged in FTD [27] and reportedly associated with both apathy and disinhibition [28, 29]. However, we did not find a direct correlation between vascular markers and neuropsychological scales, which may be due to the small sample. The integrity of the structure and function of blood vessels is key to normal brain functioning, and its dysfunction contributes to neurodegeneration [30]. Neurovascular unit dysfunction is well-established in Alzheimer’s disease [31], Parkinson’s disease [32], Huntington’s disease [33], amyotrophic lateral sclerosis [34], and multiple sclerosis [35], and researchers are developing medicines targeting the vascular pathway. Our study provides evidence for vascular dysfunction in bvFTD.

There are some limitations in our study. First, the sample size was relatively small because we enrolled a group of patients who had completed both the vascular factor test and multimodal PET/MRI. Second, our patients were clinically diagnosed with probable bvFTD without pathological verification. Third, the cross-sectional design of this study lacked longitudinal follow-up, making it impossible to identify causal relations and whether the vascular factors can be markers for prognosis. Finally, our markers are all from peripheral plasma samples. Future studies should acquire samples from both CSF and blood to provide more evidence for vascular dysfunction in the central nervous system.

## Conclusion

Abnormality of vascular function was found in patients with sporadic bvFTD, including WMH and peripheral biomarker abnormalities, which were correlated with specific brain changes of bvFTD, inflammation, and clinical measures. These results indicate vascular dysfunction and perturbance of the BBB in the pathogenesis of FTD. Further research targeting neurovascular links with FTD should be conducted, and vascular pathways may be a promising target for a potential therapy for FTD.

## Data Availability

The datasets used and analyzed during the current study are available from the corresponding author on reasonable request.

## Abbreviations

FTD: Frontotemporal dementia
bvFTD: Behavioral variant frontotemporal dementia
MRI: Magnetic resonance imaging
PET: Positron emission tomography
^18^F-FDG: ^18^F fluoro-deoxy-glucose
MMSE: Mini-Mental State Examination
CDR® plus NACC FTLD-SB: The sum of the six domains of the CDR plus the behavior/comportment/personality and language domains
BEHAV: Behavior/comportment/personality
LANG: Language
TMT: Trail-Making Test
BNT: Boston Naming Test
ADL: Activities of daily living scale
FBI: Frontal Behavior Inventory
MMP-1: Matrix metalloproteinase-1
MMP-3: Matrix metalloproteinase-3
